# Stable single atomic silver wires assembling into a circuitry-connectable nanoarray

**DOI:** 10.1038/s41467-021-21462-3

**Published:** 2021-02-19

**Authors:** Yaxin Chen, Daiming Tang, Zhiwei Huang, Xi Liu, Jun Chen, Takashi Sekiguchi, Weiye Qu, Junxiao Chen, Dongrun Xu, Yoshio Bando, Xiaolei Hu, Xiaoping Wang, Dmitri Golberg, Xingfu Tang

**Affiliations:** 1 Department of Environmental Science and Engineering, Fudan University, Shanghai, China; 2 World Premier International Center for Materials Nanoarchitectonics, National Institute for Materials Science, Namiki 1-1, Tsukuba, Ibaraki, Japan; 3 Department of Environmental Science and Engineering, College of Chemical Engineering, Huaqiao University, Xiamen, Fujian China; 4 School of Chemistry and Chemical, In-situ Center for Physical Science, Shanghai Jiao Tong University, Shanghai, China; 5 Syncat@Beijing, SynfuelsChina Co. Ltd, Beijing, China; 6 Faculty of Pure and Applied Science, University of Tsukuba, Tsukuba, Japan; 7 Australian Institute for Innovative Materials, University of Wollongong, Wollongong, NSW Australia; 8 Institute of Molecular Plus, Tianjin University, Tianjin, China; 9 Centre for Materials Science and School of Chemistry and Physics, Queensland University of Technology (QUT), Brisbane, QLD Australia; 10 Shanghai Institute of Pollution Control and Ecological Security, Shanghai, China

**Keywords:** Nanowires, Nanoscale materials

## Abstract

Atomic metal wires have great promise for practical applications in devices due to their unique electronic properties. Unfortunately, such atomic wires are extremely unstable. Here we fabricate stable atomic silver wires (ASWs) with appreciably unoccupied states inside the parallel tunnels of α-MnO_2_ nanorods. These unoccupied Ag 4*d* orbitals strengthen the Ag–Ag bonds, greatly enhancing the stability of ASWs while the presence of delocalized 5*s* electrons makes the ASWs conducting. These stable ASWs form a coherently oriented three-dimensional wire array of over 10 nm in width and up to 1 μm in length allowing us to connect it to nano-electrodes. Current-voltage characteristics of ASWs show a temperature-dependent insulator-to-metal transition, suggesting that the atomic wires could be used as thermal electrical devices.

## Abstract (web summary)

One-dimensional atomic metal wires can exhibit useful properties distinct from their bulk equivalents; however they typically suffer from limited stability. Here, Chen et al create atomic silver wires which are stable and exhibit a temperature dependent metal to insulator transition.

## Introduction

One-dimensional (1D) atomic metal wires are promising materials as building blocks in electronic nanodevices because their electronic characteristics are distinct from those of their bulk counterparts^1–5^. For practical applications of atomically thin metal wires in nanoelectronics, high stability in air at room temperature and a suitable length for convenient connection into nanocircuitry are key requirements. However, the high degree of coordinative unsaturation makes the formation of long atomic metal wires extremely challenging.

Over the past two decades, the creation of long atomic metal wires through (self-)assembly techniques have been reported only in a few cases. Atomic silver wires (ASWs) of up to 100 nm in length were synthesized on Pt(997) surfaces under ultrahigh vacuum conditions^6^. Infinite linear atomic metal wires were fabricated in the tunnels of an air-sensitive inorganic subnitride^7^. Using a scanning tunneling microscope, 1D atomic wires of gold, silver, or manganese with controllable lengths were constructed on surfaces by self-assembling^3,5,8^. However, these long atomic wires are unstable, and thus difficult to use in practical applications^7–9^. Owing to the atomically thin wire width, assembling many long atomic wires as a coherently oriented array is highly desirable for constructing miniaturized device architectures.

Herein, we create stable ASWs of up to 1 μm in length by a self-assembly technique, leading to a coherently oriented three-dimensional (3D) array inside the tunnels of an insulating α-MnO_2_ nanorod, the stability of which derives from the scaffolding function of α-MnO_2_ and the strengthened Ag-Ag bonds due to the appreciably unoccupied states of the Ag 4*d* orbitals. The stable atomic wire array with suitable 3D sizes can be conveniently connected with nanoelectrodes for conductance measurements. Current-voltage (I–V) data demonstrate a temperature-controlled insulator-to-metal transition, making ASWs attractive for application as thermal electrical devices.

## Results

### Geometric structures of the atomic wire array

ASWs were synthesized by a thermal diffusion method starting from Ag nanoparticles (NPs) supported on surfaces of the α-MnO_2_ nanorods (see the “Methods” section for more details). The accurate geometrical structures of ASWs were investigated by synchrotron X-ray diffraction (SXRD) patterns, extended X-ray absorption fine structure (EXAFS) spectroscopy and transmission electron microscopy (TEM) imaging. TEM data shows that the incorporation of Ag atoms hardly changed the morphology of α-MnO_2_ nanorods (Supplementary Figs. 1 and 2). We further conducted a Rietveld refinement of room-temperature SXRD of ASWs inside the α-MnO_2_ tunnels together with the pristine α-MnO_2_ (Supplementary Fig. 3)^10^. The resulting lattice information and structural parameters were summarized in Supplementary Tables 1 and 2. These data indicate that the Ag atoms of ASWs are located at the Wychoff 2a sites inside the α-MnO_2_ tunnels^11^, and thus the closest Ag–Ag and Ag–O distances are determined to be 2.87  and 2.48 Å, respectively.

**Fig. 1:**
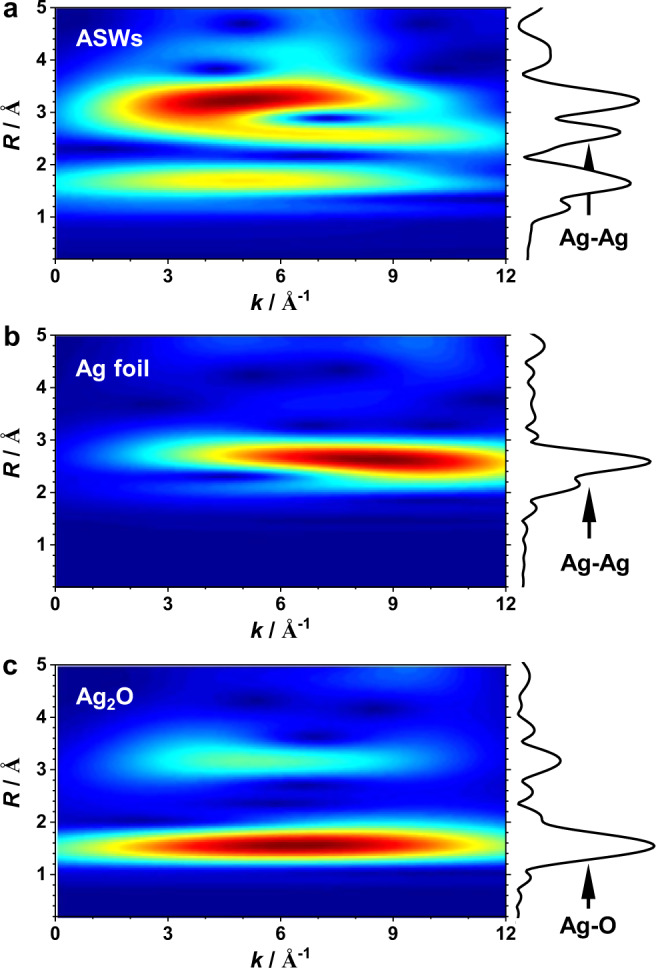
Local structures of Ag atoms in ASWs. Wavelet transform plots and *k*^3^-weighted EXAFS spectra in *R*-space of Ag (**a**), ASWs (**b**), and Ag_2_O (**c**). Color scale: dark blue to red refers to low intensity to high intensity.

The local structure of Ag atoms in ASWs was further explored by EXAFS spectra at the Ag *K*-edge using both Fourier and wavelet transforms (Fig. 1). The wavelet transform plots show that the maxima of the wavelet transforms of Ag foil and Ag_2_O are ~8 and ~7 Å^−1^, which correspond to Ag–Ag and Ag–O scattering paths, respectively. Compared with the two references, ASWs have *k* maxima at ~5, ~6, and ~7 Å^−1^, which should correspond to the first-shell Ag–O, metallic Ag–Ag, and second-shell Ag–O/Mn scattering paths, respectively. To verify this inference, we conducted EXAFS fitting to unveil the quantitative coordination configuration of Ag atoms. The fitting results show that in ASWs the interatomic distances in the two nearest neighbor shells are attributed to Ag–O (~2.48 Å) and Ag–Ag (~2.87 Å) bonds with coordination numbers (CNs) of 4 and 2, respectively (Supplementary Fig. 4 and Table 3). These findings agree with the data of the SXRD refinements above. The average Ag–Ag bond length is close to that of 2.89 Å in bulk Ag (Supplementary Fig. 5 and Table 3)^12^, implying that there is a strong Ag–Ag bonding in an ASW. The shortest Ag–O distance of 2.48 Å is significantly longer than the Ag–O bond length of 2.04 Å in Ag_2_O (Supplementary Fig. 6 and Table 3), indicative of a weak interaction between ASWs and α-MnO_2_.

**Fig. 2:**
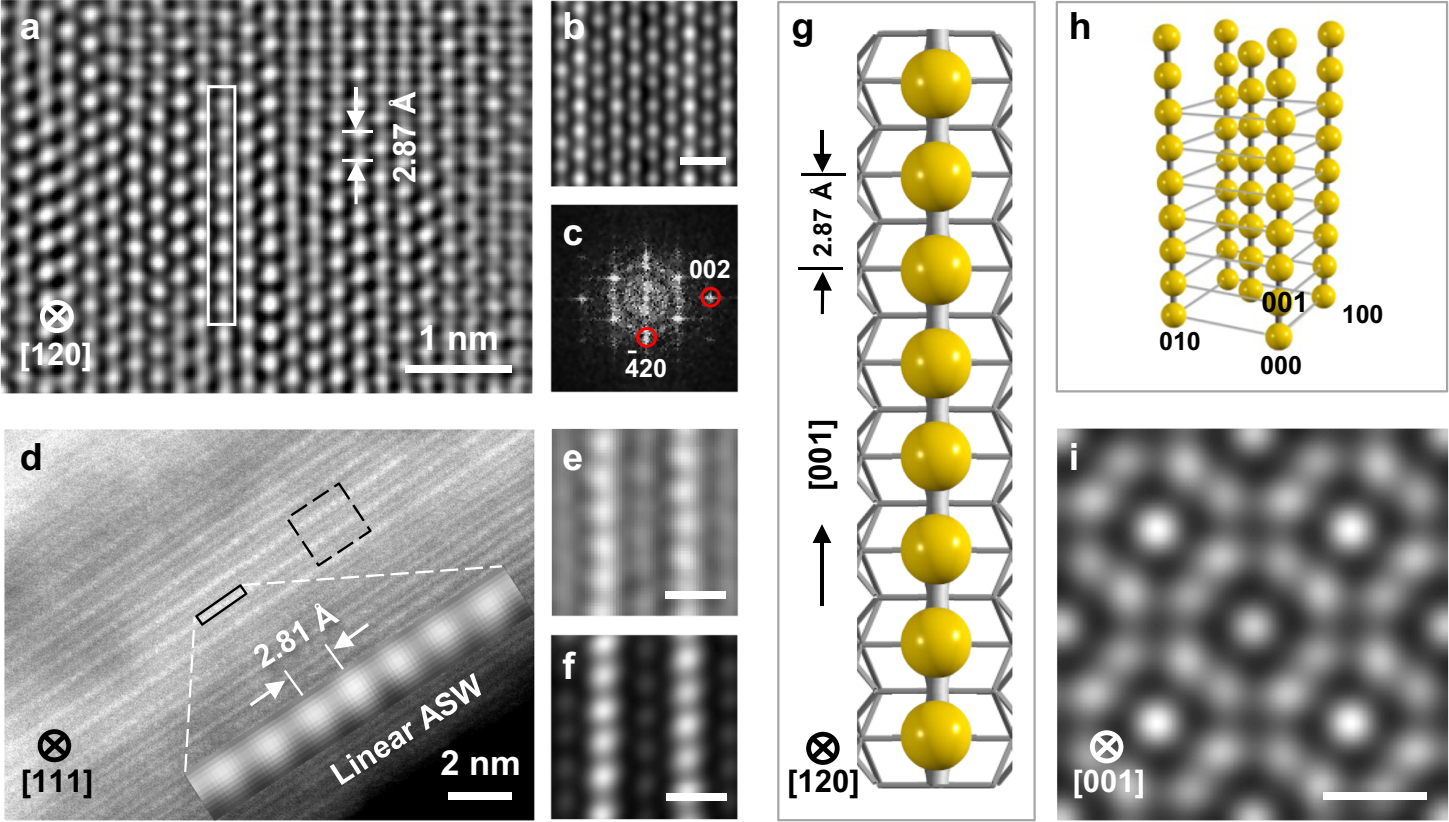
Electron microscopy imaging and structure models of ASWs. **a**, **b** Filtered and simulated HRTEM images viewed from a α-MnO_2_[120] axis. **c** Calculated diffraction pattern from **a**. **d** HAADF-STEM image, viewed from the α-MnO_2_[111] direction (the inset shows an enlarged linear Ag wire after Fourier filtering). **e**, **f** Filtered and simulated images enlarged from the black dashed rectangle in **d**. **g** Structural model of one typical ASW together with the α-MnO_2_ framework. **h** 3D model of the array with five ASWs. **i** Simulated image of ASWs in the α-MnO_2_ tunnels viewed from the [001] direction showing the inter-wire distance. Scale bars in **b**, **e**, **f**, and **i**, 0.5 nm.

The geometric structures of ASWs were directly imaged by high-resolution TEM (HRTEM). Figure 2a–c shows an HRTEM image, a simulated image^13,14^, and a calculated diffraction pattern of ASWs viewed along an α-MnO_2_[120] axis, respectively. In Fig. 2a, ASWs along the α-MnO_2_[001] direction with the Ag–Ag bond length of ~2.87 Å are clearly visible, approaching the typical Ag–Ag bond length in bulk Ag. Similarly, parallel wires of bright atomic columns are observed along the α-MnO_2_[111] direction in the high angle annular dark field scanning transmission electron microscopy (HAADF-STEM) image (Fig. 2d), particularly shown in the images after Fourier filtering (Fig. 2d inset and Fig. 2e)^14^. As the scattering cross section in HAADF-STEM image is approximately proportional to Z^1.8^ (where Z is the atomic number)^15^, Ag atoms appear brighter than Mn and O atoms. The bright linear atomic columns in the HAADF-STEM image thus are ASWs, as shown by a corresponding simulated image (Fig. 2f). An observed distance of ~2.81 Å for a typical ASW is attributed to the projected distance of the Ag–Ag bond length of ~2.87 Å on the α-MnO_2_(111) plane. These observations are in good accordance with the results of SXRD pattern and EXAFS spectra.

A structural model of the linear atomic wires was constructed on the basis of the above results (Fig. 2g). According to the experimental design, we filled all tunnels of the α-MnO_2_ nanorods with Ag atoms to form the linear atomic Ag wires (Supplementary Fig. 2). As a result, the average length of ASWs is estimated to be ~0.5 μm with ~4% of ASWs longer than 1 μm (Supplementary Fig. 2). The average width of the α-MnO_2_ nanorods is ~12 nm, and more than 280 parallel ASWs were assembled in the tunnels of each α-MnO_2_ nanorod to form an atomic wire array (Fig. 2h). The closest-neighbor inter-wire spacing of this array is 6.95 Å (Fig. 2i). Hence, the suitable 3D sizes of this array enable ASWs as one electrical device to be connected into a circuit.

### Assembly process of the atomic wire array

**Fig. 3:**
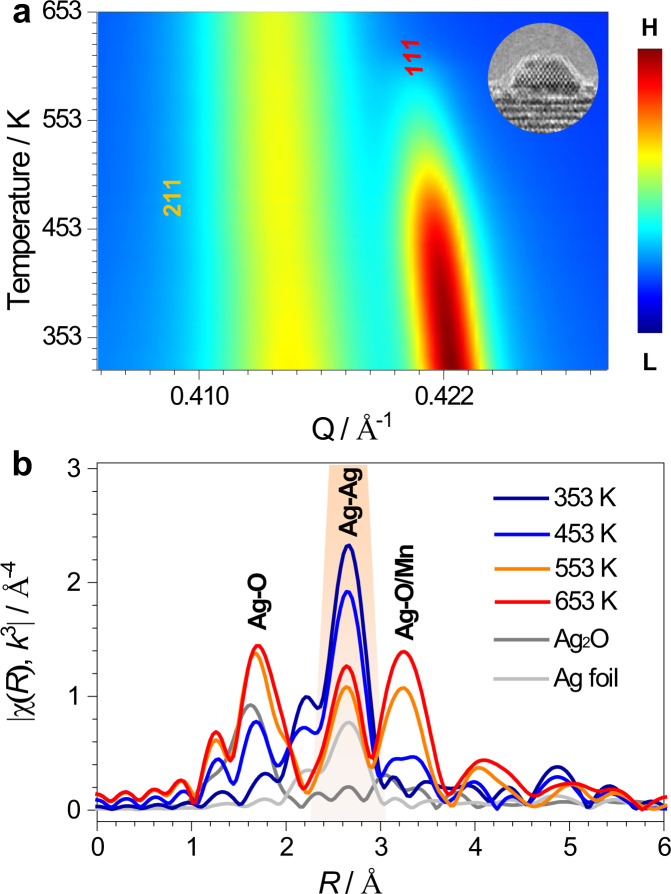
Assembly process of ASWs. **a** Contour map of temperature-programmed SXRD patterns of Ag/MnO_2_ as a function of momentum transfer (Q) from 323 to 653 K, showing the diffraction intensities of the Ag(111) and the α-MnO_2_(211) reflections. Inset: HRTEM image of Ag/MnO_2_ with a Ag NP of 2 nm in height and 4 nm in width. H and L in the scale bar refer to High and Low in intensity, respectively. **b** Room-temperature ex situ Ag *K*-edge *χ*(R) *k*^3^-weighted FT EXAFS spectra of Ag/MnO_2_ after being annealed at higher temperatures, and two references of Ag_2_O and Ag foil. The amplitudes of Ag_2_O, Ag foil, and Ag/MnO_2_ spectra after being annealed at 353 K and 453 K were multiplied by 1/4, 1/18, 1/4, and 2/3, respectively.

Next we present the assembly process of ASWs. We firstly prepared α-MnO_2_ nanorods of up to 1 µm in length and over 10 nm in width, which have square 1D parallel tunnels extending along the [001] growth direction^16^. Ag/MnO_2_ was then synthesized by depositing truncated octahedral Ag NPs onto the α-MnO_2_ surfaces (inset of Fig. 3a, Supplementary Fig. 7). Normally, supported metal NPs are prone to sintering and forming larger particles through thermal activation^17,18^ via Ostwald ripening and/or coalescence^19^, depending on the particle size^20^. In our work, however, shrinking of Ag NPs on the α-MnO_2_ surfaces was clearly observed by using temperature-programmed SXRD technique, in situ TEM, and EXAFS spectroscopy.

Figure 3a shows the temperature-programmed SXRD patterns of Ag/MnO_2_. The intensity of the SXRD pattern of Ag NPs becomes weaker and ultimately disappears as the temperature increases, indicating that Ag NPs gradually shrink and finally reach a highly dispersive state. In particular, the Ag(111) reflection fades as the annealing temperature increases. At 383 K, Ag atoms become sufficiently active, detaching from Ag NPs and diffusing along the α-MnO_2_ surfaces (Supplementary Fig. 8), similar to what has been observed for Ag atoms diffusing across Pt-Ag step boundaries^6^. As the temperature further increases to ~493 K, a clear anomaly in the first derivative SXRD patterns is observed (Supplementary Fig. 8), implying that the Ag atoms start to be assembled into the α-MnO_2_ tunnels.

Recent work shows that the reactive environment plays a key role in the redispersion of noble metal NPs^21^. In order to understand the mechanism behind the novel process and rationalize the influence of atmospheres, we use an advanced in situ environmental TEM tool to record the structural evolution of MnO_2_ supported Ag NPs in situ and with high spatial resolutions (Supplementary Figs. 9–10). Clearly, the annealing under the inert environment cannot trigger the re-dispersion of Ag NPs, but causes the serious aggregation. In contrast, the re-dispersion of Ag NPs took place during the oxygen annealing, leading to the formation of ASWs. This result keeps a good agreement with the temperature-programmed SXRD data. The in situ environmental TEM results evidence that the interaction between Ag NPs and O_2_ is the driving force to enhance the mobility of Ag atoms^22^, which move from the NPs to the support and are finally hosted in the matrix^23–25^. As showed in Supplementary Fig. 11 and corresponding Supplementary Movie, a Ag NP collapsed and quickly disappeared in 5 min at 270 °C in the presence of O_2_. During the redispersion process, a stronger adherence of Ag NP to α-MnO_2_ was observed, leading to the collapse of the Ag NP. It looks like that the process is surface-mediated, in which atomic species after being emitted from a metal NP diffuse on the surface of the support until being trapped by a strong metal-support interaction^23–25^ (Supplementary discussion).

The local structures of the Ag atoms during the thermal diffusion process were determined by using in situ EXAFS spectroscopy (Supplementary Fig. 12). To eliminate the effects of non-symmetric and inhomogeneous distribution of the instantaneous bond length owing to the thermal disorder^26^, the corresponding ex situ EXAFS spectra were measured after the samples were annealed at higher temperatures and then cooled down to room temperature (Fig. 3b). As the annealing temperature increases, the Fourier transform (FT) amplitude due to the first Ag–Ag shell decreases, while the FT amplitudes due to two Ag–O shells increase. Specifically, after annealing at 653 K, the Ag atoms have been assembled into the tunnels to form ASW arrays. Although the geometric size of the α-MnO_2_ tunnel is 4.7 Å × 4.7 Å, the inner effective diameter is so small that it only allows single Ag atoms to diffuse along the tunnel at one time^27,28^. Thus, the atomic wire arrays are formed via the atom-by-atom assembly process.

### Electronic and electrical properties of the atomic wire array

**Fig. 4:**
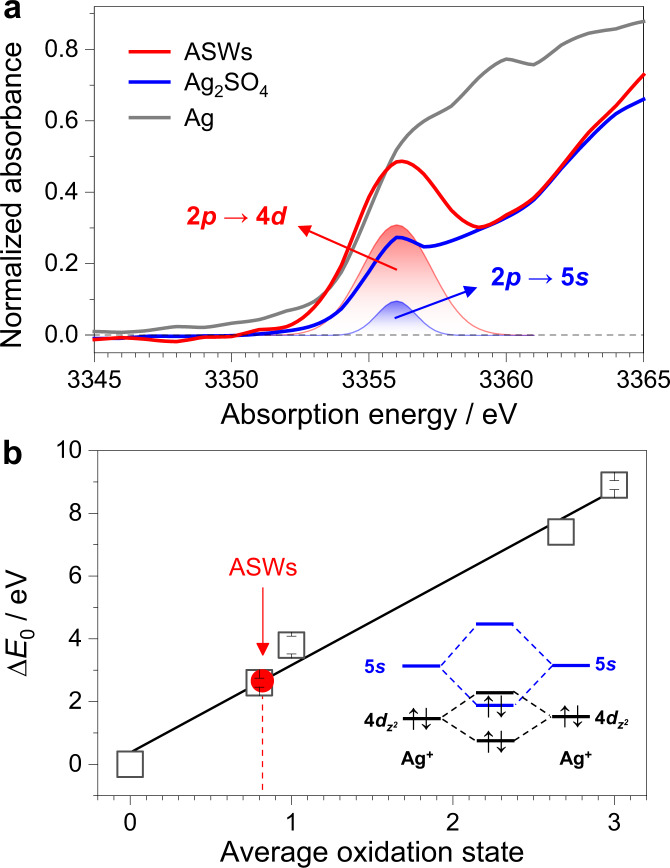
Electronic structures of ASWs. **a** XANES spectra of ASWs and two references at the Ag *L*_3_-edge. Red and blue shades represent the edge peaks of ASWs and Ag_2_SO_4_, respectively. **b** The change of Ag *L*_1_-edge energy (∆*E*_0_) versus average oxidation state. The Ag metal edge energy is selected as the reference energy, i.e., ∆*E*_0_ = 0 eV. Data of binary silver oxides containing Ag in formal oxidation states of +0.8, +1, +2.67, and +3 were obtained from reference ^31^. Inset: Schematic illustration of the 4*d*–5*s* hybridization or overlapping among Ag atoms of ASWs leading to the 5*s* bonding orbitals shifting down below the 4*d* anti-bonding orbitals^15^.

Finally, we explored the electronic structures and conducting properties of ASWs. Figure 4a shows the X-ray absorption near edge structure (XANES) spectra of ASWs, metallic Ag powder and Ag_2_SO_4_ at the Ag *L*_3_ edge. Metallic Ag powder and Ag_2_SO_4_, respectively with the Ag^0^(4*d*^10^5*s*^1^) and Ag^+^(4*d*^10^5*s*^0^) electronic configuration are chosen as references in order to more precisely determine the electronic structures of ASWs^15^. No distinct peak at ∼3354 eV appears in the XANES spectrum of metallic Ag because of the absence of the unoccupied 4*d* orbitals (4*d*^10^). A weak peak at 3354.8 eV was observed in the spectrum of Ag_2_SO_4_, which should be assigned to the 2*p*_3/2_ → 5 *s* transition and enhanced by the 4*d*–5*s* hybridization according to the fitting analysis (Fig. 4a)^15^. At the same absorption energy of 3354.8 eV, a strong peak appearing in the XANES spectrum of ASWs was mainly attributed to dipole-allowed 2*p*_3/2_ → 4*d* transitions, suggesting the presence of the unoccupied Ag 4*d* orbitals^15^. The unoccupied orbitals are characteristics of anti-bonding orbitals, thus reinforcing Ag–Ag bonds^29,30^. Therefore, the reinforced Ag–Ag bonds together with the scaffolding function of α-MnO_2_ via the weak Ag–O interactions enable ASWs to be very stable in air.

The electronic states of ASWs were further studied by using the XANES spectroscopy at the Ag *L*_1_ edge (Supplementary Fig. 13), which can precisely determine the oxidation states^31^. Figure 4b shows the differential edge energies (∆*E*_0_) with respect to that of metallic Ag as a function of average oxidation states. Thus, the average oxidation state of ASWs was determined to be close to +1 (more precisely +0.82) according to the calibrated curve. On the basis of the results in Fig. 4a, the strong intensity of the edge peak reflects the hole in the 4*d* orbitals. By combining the results of XANES spectra at the Ag *L*_3_ and *L*_1_ edges, we deduced that ASWs might have a Ag^+0.8^ (4*d*^9.2^5*s*^1^) electronic configuration. This evidence implies the presence of the 4*d*–5*s* hybridization, allowing the energy of the 5 *s* bonding orbitals to be lower than that of the 4*d* anti-bonding orbitals. It is also not difficult to interpret this configuration by using a molecular orbital theory^15,31^. As calculated by Behrens^31^, if the filled Ag $4d_{z^{2}}$ and empty 5*s* orbitals have the same symmetry and are hybridized, the 4*d* vacancy concomitant with the 5*s* occupied orbital is created (inset of Fig. 4b). As for ASWs, we set the wire direction as the *Z* axis, and thus $4d_{z^{2}}$ and 5*s* orbitals have the same symmetry, resulting in the $4d_{z^{2}}$ vacancy. In principle, the 4*d* and 5*s* electrons are often characteristics of localized and delocalized features, respectively^32^. As a consequence, the depletion of the occupied states of the $4d_{z^{2}}$ anti-bonding orbitals strengthens the Ag–Ag bonds along the wire direction, greatly enhancing the stability of ASWs, and owing to the orbital spherical symmetry, the presence of the 5*s* electrons with the delocalized feature endows ASWs with the conducting property.

**Fig. 5:**
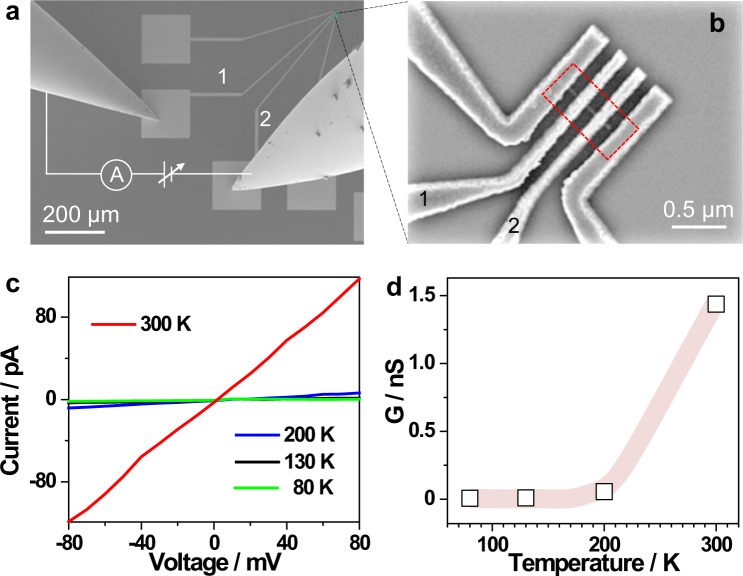
Electrical properties of ASWs. **a** Circuit setup for the I–V measurements of ASWs. **b** An ASW array bridged between the nanoelectrodes (labeled as 1 and 2, highlighted in a red dashed rectangle), which was enlarged from the green rectangle in **a**. **c** The I–V curves of the ASW at different temperatures. **d** The corresponding conductance (G) of the array calculated from the slopes of the I–V curves (**c**).

We measured the I-V curves of metallic ASWs in a temperature range of 80–300 K by bridging one typical atomic wire array between two nanoelectrodes to make an electrical circuit device (Fig. 5a, b). Figure 5c,d show the I–V curves of ASWs at different temperatures and the corresponding conductance as a function of temperature, respectively. ASWs exhibit thermally sensitive conductive properties of a temperature-dependent insulator-to-metal transition. Below 200 K, the current through ASWs is close to zero in the applied voltage range, and thus the conductance of ASWs approaches zero (Fig. 5d), implying an insulating behavior, i.e., the device is switched off. At 300 K, the linear I–V relation of ASWs demonstrates the Ohmic conductance (Fig. 5c) with a value of 1.5 nS (Fig. 5d) calculated from the slope of the red I–V curve in Fig. 5c, and, thus, the device is switched on. The I–V curve measured at 300 K confirms that electrons can be transferred along ASWs. Therefore, owing to the insulator-to-metal transition feature, ASWs can switch ON and OFF a circuit device, and this electrical feature allows the atomic wire array to be used as new thermal electrical devices.

## Discussion

To understand possible mechanisms behind the atomic assembling, we carried out the O 1*s*, Mn 2*p* X-ray photoelectron spectra (XPS) of the atomic wire array. Supplementary Figure 14 depicts O 1*s* XPS of Ag/MnO_2_ and ASWs in the α-MnO_2_ tunnels together with their difference, and only a little discrepancy in their O 1*s* XPS is discernible. Subtly, after Ag NPs were transferred into ASWs in the α-MnO_2_ tunnels, the intensity of the O 1*s* XPS of MnO_2_ slightly decreases in 532–534 eV, and increases at 530.5 eV, suggesting that the electron density of the O atoms slightly increases after the formation of the atomic wire array in the α-MnO_2_ tunnels. The oxidation states of Mn are preserved after the formation of ASWs (Supplementary Fig. 15). This indicates that the interaction mainly occurs between O atoms of α-MnO_2_ and Ag of ASWs with a small amount of the Ag 4*d* electrons transferring to the O atoms of α-MnO_2_, resulting in the partially unoccupied Ag 4*d* states, consistent with the results obtained from the XANES spectra above. The partial depletion of the electronic states is found at the top of the 4*d* orbitals with anti-bonding character^33^, implying an increased strength of the Ag–Ag bonds^34^. As a result, α-MnO_2_ has three main functions: (i) as a scaffold to protect ASWs from coalescence, (ii) as a special electron acceptor to only deplete the occupied states of Ag 4*d* anti-orbitals to strengthen the Ag–Ag bonds and keep the Ag 5 *s* electrons with the delocalized states, and (iii) as an ideal container to assemble ASWs into the coherently oriented array with the suitable 3D sizes, which enables the array to be connected into thermal electrical devices. In fact, α-MnO_2_ is not unique in the family of the porous materials, and we anticipate that other metal atomic wires or arrays can also be created by choosing suitable porous materials and optimizing host-guest interactions.

## Methods

### Sample synthesis

All the chemicals are of analytical grade and used as received.

### Synthesis of ASWs

α-MnO_2_ was prepared by a hydrothermal route with an aqueous solution of MnSO_4_ (0.20 mol L^−l^), (NH_4_)_2_S_2_O_8_ (0.20 mol L^−l^), and (NH_4_)_2_SO_4_ (1.00 mol L^−l^) at 393 K for 12 h. The obtained sample was filtered, washed with deionized water, dried at 383 K for 24 h, and calcined at 673 K for 4 h. The sample is expressed as A_*x*_Mn_8_O_16_ (*x* ≤ 2)^28^, where A denotes the tunnel sites of α-MnO_2_. AgNO_3_ (0.733 g) was dissolved in de-ionized water to get a solution (60 mL) at room temperature, to which an aqueous ammonia solution (25 wt.%) was slowly added under stirring until the solution became transparent. Then, both the transparent solution and an H_2_O_2_ solution (30 wt.%, 30 mL) were simultaneously added to another suspension (80 mL) containing α-MnO_2_ (1.760 g) under stirring at 298 K for 1 h. The final suspension was filtered, washed with deionized water, and then dried in 353 K for 24 h to get Ag NPs supported on α-MnO_2_ surfaces. Ag/MnO_2_ was annealed at 653 K in air for 4 h to obtain ASWs inside α-MnO_2_ tunnels.

### Transmission electron microscopy (TEM) images

TEM, high resolution TEM (HRTEM) images, and energy-dispersive X-ray (EDX) microanalyses (point/linescan analyses) were carried out with a JEOL JEM-2100F field-emission gun transmission electron microscope operated at an accelerating voltage of 200 kV and equipped with an ultra-high resolution pole-piece that provides a point-resolution better than 0.19 nm. It was also equipped with a STEM control unit (Gatan), EDX detector (SDD 80 mm^2^), CCD camera (14-bit Gatan Orius SC600), bright-field (BF), and HAADF detectors (JEOL). Some microscope parameters used were as follows: defocus: −43.4 nm, Cs (spherical aberration): 0.5 mm, Cc (chromatic aberration): 1.0 mm. Fine powders of the materials were dispersed in ethanol, sonicated, and sprayed on a carbon coated copper grid, and then allowed to air-dry. Gatan SOLARUS 950 was used before observation. TEM simulations were conducted using the QSTEM v2.22 software based on multi-slice algorithm. A plane wave incident on the sample is modified by the projected potential of an atomic model partitioned into different slices. The modified complex wave is propagated through each slice of the sample.

### In situ TEM image

We used an in situ gas holder (Climate S3, DENSsolutions) and a dedicated field-emission S/TEM (FEI Talos F20X) with an accelerating voltage of 200 kV to conduct the in-situ annealing experiments under different gaseous environments. The as-prepared Ag NPs supported on α-MnO_2_ (Ag/MnO_2_) was used as a precursor. The sample was directly dispersed in a MEMS chip for the in situ TEM experiment. After the holder equipped with the chip was inserted into TEM, different gases, N_2_ or O_2_, was introduced into the holder and the inner pressure was kept at 1 atm pressure. The temperature was gradually increased from room temperature to desired temperatures. Once the temperature reached at setting points, HAADF-STEM image of the sample was recorded. In order to reduce beam damage, electron beam was completely closed during the annealing treatment and the dose rate was minimized. Clearly, the imaging conditions allow for no damages to both Ag NPs or α-MnO_2_. We also recorded the dynamic redispersion process of the supported Ag nanoparticles at 270 °C under atmospheric oxygen to visualize the deconstruction of the selected nanoparticles.

### Synchrotron X-ray diffraction (SXRD) patterns

The room temperature SXRD patterns were recorded at BL14B of the Shanghai Synchrotron Radiation Facility (SSRF) at a wavelength of 0.6883 Å. A Mar345 image plate detector was employed for the data collection and the data were further integrated using the fit2d code^35^. The beam was monochromatized using a Si(111) crystal and a Rh/Si mirror was used for the beam focusing to a size of ~0.5 × 0.5 mm^2^. Rietveld refinements of the diffraction data were performed with the FULLPROF software package on the basis of the space group *I*4/*m*. The temperature-programmed SXRD patterns were collected at BL14B of SSRF at a wavelength of 1.2398 Å. A typical amount of the sample (~1.5 mg) was loaded in a quartz capillary tube with a diameter of ~1 mm, and then heating was carried out using a temperature-programmed procedure at a ramp of 3 K min^−1^. Each SXRD pattern was collected at 3-min intervals and analyzed by using a CMPR software.

### X-ray absorption spectra

X-ray absorption spectra at the Ag *K*-edge were measured at BL14W of the SSRF with an electron beam energy of 3.5 GeV and a ring current of 200–300 mA. The data were collected with a fixed exit monochromator using two flat Si(311) crystals. Harmonics were rejected by using a grazing incidence mirror. The XANES spectra at the Ag *L*_1_,_3_-edge were acquired at BL4B7A of the Beijing Synchrotron Radiation Facility (BSRF) with an electron beam energy of 2.2 GeV and a ring current of 300–450 mA. The energy step for XANES measurement was set to be 0.2 eV. The extended X-ray absorption fine structure (EXAFS) spectra were collected in a transmission mode using ion chambers filled with N_2_. The raw data were analyzed by using the IFEFFIT 1.2.11 software package.

### X-ray photoelectron spectra (XPS)

XPS were recorded using Kratos Axis Ultra-DLD system with a charge neutralizer and a 150 W Al (Mono) X-ray gun (1486.6 eV) equipped with a delay-line detector (DLD). The spectra were acquired at a normal emission with a passing energy of 40 eV. The spectra were calibrated according to the C 1*s* peak at 284.6 eV.

### Electrical measurements by in situ scanning electronic microscopy (SEM) technique

Samples were dispersed in ethanol and dropped on a marked Si substrate with 300 nm SiO_2_ layer. The position of the suitable individual wire array was determined by SEM observations (JSM-6700F). The Cr/Au (10 nm/100 nm) electrodes were patterned onto the top of the atomic wire array using lithography and electron-beam deposition followed by a lift-off process. Then, the wafer was transferred into a SEM with piezoelectric micromanipulators (Kleindiek). Finally, temperature-dependent electrical measurements were carried out by using a semiconductor characterization system (Keithley 4200) and a home-made cooling stage.

## Supplementary information

Supplementary Information

Supplementary Movie

Peer Review File

Description of Additional Supplementary Files

## Data Availability

All the data that support the findings of this study are available from the corresponding author upon reasonable request. Source data are provided with this paper.

## Source data

Source Data

## References

[1] Yanson AI, Bollinger GR, van den Brom HE, Agrait N, van Ruitenbeek JM (1998). Formation and manipulation of a metallic wire of single gold atoms. Nature.

[2] Ohnishi H, Kondo Y, Takayanagi K (1998). Quantized conductance through individual rows of suspended gold atoms. Nature.

[3] Nilius N, Wallis TM, Ho W (2002). Development of one-dimensional band structure in artificial gold chains. Science.

[4] Crain JN, Pierce DT (2005). End states in one-dimensional atom chains. Science.

[5] Chen C, Bobisch CA, Ho W (2009). Visualization of Fermi’s golden rule through imaging of light emission from atomic silver chains. Science.

[6] Gambardella P, Blanc M, Brune H, Kuhnke K, Kern K (2000). One-dimensional metal chains on Pt vicinal surfaces. Phys. Rev. B..

[7] Höhn P (2006). (Ca_7_N_4_)[M_x_](M= Ag, Ga, In, Tl): Linear metal chains as guests in a subnitride host. Angew. Chem. Int. Ed..

[8] Hirjibehedin CF, Lutz CP, Heinrich AJ (2006). Spin coupling in engineered atomic structures. Science.

[9] Rodrigues V, Fuhrer T, Ugarte D (2000). Signature of atomic structure in the quantum conductance of gold nanowires. Phys. Rev. Lett..

[10] Rietveld HM (1969). A profile refinement method for nuclear and magnetic structures. J. Appl. Cryst..

[11] Ling C, Mizuno F (2012). Capture lithium in αMnO_2_: Insights from first principles. Chem. Mater..

[12] Moon HR, Choi CH, Suh MP (2008). A stair-shaped molecular silver(0) chain. Angew. Chem. Int. Ed..

[13] Koch, C. *Determination of Core Structure Periodicity and Point Defect Density Along Dislocations*. PhD thesis. Arizona State University (2002).

[14] Koch C, Spence JCH, Zorman C, Mehregany M, Chung J (2000). Modelling of HREM and nanodiffraction for dislocation kinks and core reconstruction. J. Phys. Condens. Mat..

[15] Miyamoto T, Niimi H, Kitajima Y, Naito T, Asakura K (2010). Ag *L*_3_-edge X-ray absorption near-edge structure of 4d_10_ (Ag^+^) compounds: Origin of the edge peak and its chemical relevance. J. Phys. Chem. A.

[16] Huang Z (2012). Catalytically active single‐atom sites fabricated from silver particles. Angew. Chem. Int. Ed..

[17] Farmer JA, Campbell CT (2010). Ceria maintains smaller metal catalyst particles by strong metal-support bonding. Science.

[18] Lei Y (2010). Increased silver activity for direct propylene epoxidation via subnanometer size effects. Science.

[19] Bowker M (2002). The going rate for catalysts. Nat. Mater..

[20] Campbell CT, Parker SC, Starr DE (2002). The effect of size-dependent nanoparticle energetics on catalyst sintering. Science.

[21] Van Deelen TW, Mejía CH, de Jong KP (2019). Control of metal-support interactions in heterogeneous catalysts to enhance activity and selectivity. Nat. Catal..

[22] Gänzler AM (2017). Tuning the structure of platinum particles on ceria in situ for enhancing the catalytic performance of exhaust gas catalysts. Angew. Chem. Int. Ed..

[23] Yao Y (2019). High temperature shockwave stabilized single atoms. Nat. Nanotechnol..

[24] Lang R (2019). Non defect-stabilized thermally stable single-atom catalyst. Nat. Commun..

[25] Liu K (2020). Strong metal-support interaction promoted scalable production of thermally stable single-atom catalysts. Nat. Commun..

[26] Bordiga S, Groppo E, Agostini G, van Bokhoven JA, Lamberti C (2013). Reactivity of surface species in heterogeneous catalysts probed by in situ X-ray absorption techniques. Chem. Rev..

[27] Kijima N, Ikeda T, Oikawa K, Izumi F, Yoshimura Y (2004). Crystal structure of an open-tunnel oxide α-MnO_2_ analyzed by Rietveld refinements and MEM-based pattern fitting. J. Solid State Chem..

[28] Chang FM, Jansen M (1984). Ag_1.8_Mn_8_O_16_: Square planar coordinated Ag^⊕^ ions in the channels of a novel hollandite variant. Angew. Chem. Int. Ed..

[29] Thijssen WHA, Strange M, de Brugh JMJA, van Ruitenbeek JM (2008). Formation and properties of metal-oxygen atomic chains. N. J. Phys..

[30] Thijssen, W. H. A. *Molecule-assisted Atomic Chain Formation*. PhD thesis, Leiden University (2007).

[31] Behrens P, Aβmann S, Bilow U, Linke C, Jansen M (1999). Electronic structure of silver oxides investigated by AgL XANES spectroscopy. Z. Anorg. Allg. Chem..

[32] Goh J, Akola J (2015). Superatom model for Ag–S nanocluster with delocalized electrons. J. Phys. Chem. C..

[33] Thijssen WHA, Marjenburgh D, Bremmer RH, van Ruitenbeek JM (2006). Oxygen-enhanced atomic chain formation. Phys. Rev. Lett..

[34] Smit RHM, Untiedt C, Yanson AI, van Ruitenbeek JM (2001). Common origin for surface reconstruction and the formation of chains of metal atoms. Phys. Rev. Lett..

[35] Hammersley AP, Svensson SO, Hanfland M, Fitch AN, Hausermann D (1996). Two-dimensional detector software: from real detector to idealised image or two-theta scan. High. Press. Res..

