# Supplementary material for: Stable single atomic silver wires assembling into a circuitry-connectable nanoarray
Source: Nat Commun. 2021 Feb 19;12:1191. doi: 10.1038/s41467-021-21462-3 (PMC7895918; doi:10.1038/s41467-021-21462-3)
Supplement: Supplementary file 1 — Supplementary Information [file 41467_2021_21462_MOESM1_ESM.pdf]

Supplementary Information

Stable single atomic silver wires assembling into a circuitry-connectable nanoarray

Yaxin Chen, Daiming Tang, Zhiwei Huang, Xi Liu, Jun Chen, Takashi Sekiguchi, Weiye Qu, Junxiao Chen, Dongrun Xu, Yoshio Bando, Xiaolei Hu, Xiaoping Wang, Dmitri Golberg & Xingfu Tang

Contents

Supplementary Discussion **3**

1.1. Dispersion mechanism 3

Supplementary Figures **5**

Figure S1. Morphology of α-MnO_2 5

Figure S2. Morphology and EDX analysis of ASWs 6

Figure S3. SXRD patterns of the α-MnO_2 and ASWs inside the α-MnO_2 tunnels 7

Figure S4. Ag *K*-edge X-ray absorption spectrum and the fitting curves of ASWs 8

Figure S5. Ag *K*-edge X-ray absorption spectrum and the fitting curves of Ag foil 9

Figure S6. Ag *K*-edge X-ray absorption spectrum and the fitting curves of Ag_2O 10

Figure S7. TEM image of Ag/MnO_2 11

Figure S8. Temperature-programmed SXRD patterns of Ag/MnO_2 12

Figure S9. In situ HAADF-STEM images of Ag/MnO_2 recorded at different temperatures during the heating treatment under atmospheric N_2 environment 13

Figure S10. In situ HAADF-STEM images of Ag/MnO_2 recorded at different temperatures during the heating treatment under atmospheric O_2 environment 14

Figure S11. A serial of in situ TEM images of Ag/MnO_2 recorded at 270 ^{o}C in the presence of O_2 15

Figure S12. In situ Ag *K*-edge χ(R) k^3-weighted FT EXAFS spectra of Ag/MnO_2 16

Figure S13. Ag L_1-edge XANES spectra and first derivative spectra of samples 17

Figure S14. O 1*s* XPS of Ag/MnO_2 and ASWs in the α-MnO_2 tunnels together with their difference 18

Figure S15. Mn 2*p* XPS of Ag/MnO_2 and ASWs in the α-MnO_2 tunnels together with their difference 19

Figure S16. The change of the Ag nanoparticles as a function of calcination time and temperature 20

Supplementary Tables **21**

Table S1. Crystallographic data and details of the α-MnO_2 and the ASWs in the α-MnO_2 pores in the data collections and the Rietveld refinements 21

Table S2. Structural parameters of the α-MnO_2 and the ASWs in the α-MnO_2 pores 22

Table S3. EXAFS parameters of ASWs, Ag foil, and Ag_2O 23

1. Supplementary Discussion

1.1 Dispersion mechanism

As showed in Supplementary Figure 11 and corresponding Movie S1, a Ag nanoparticle collapsed and quickly disappeared in 5 minutes. It suggests that many tools, including scanning/transmission electron microscope (S/TEM), scanning tunneling microscope (STM) and X-ray absorption spectroscopy (XAS), cannot identify this dynamic process promptly. In order to understand the mechanism related with the re-dispersion process, we alternatively analyze the collapse of Ag nanoparticles under the reaction conditions by using in situ XRD and environmental TEM techniques. Therefore, the decrease in volume of a single Ag nanoparticle as a function of calcination time under the reaction conditions was displayed in Supplementary Figure 16a. Clearly, the shrink of the nanoparticle size could be distinguished into three distinctive stages: initially, the nanoparticle's volume decreased slowly with the particle size larger than about 13 nm; afterwards, the nanoparticle's volume dropped rapidly with the particle size ranging from 10 to 13 nm; when the particle was smaller than 10 nm, the shrink became slower until the whole particle disappeared. It suggests that the re-dispersion process might be modulated by multi factors, which played different roles at these stages. Presumably, when the particle size became smaller, the strong metal-support interaction (SMSI) may play a main role to hinder the immigration of Ag atoms out of the parent particle. A similar trend was also observed in the in situ XRD analysis as shown in Supplementary Figure 16b, which indicates the change in Ag_{111} peak area as a function of calcination temperature. Supplementary Figures 9-10 rationalize that the main driving force of the redispersion is the interaction between Ag atoms and molecular oxygen. However, the HRTEM image of the intermediates suggests that the process is surface-mediated, in which surface oxygen atoms also interact with detached Ag atoms and modulate their transportation. Apparently,

when the destruction rate of Ag nanoparticles was not constant and changed in different circumstances related with particle sizes and the interaction between the support and NPs, the growth parameters for ASWs would change consequently.

2. Supplementary Figures

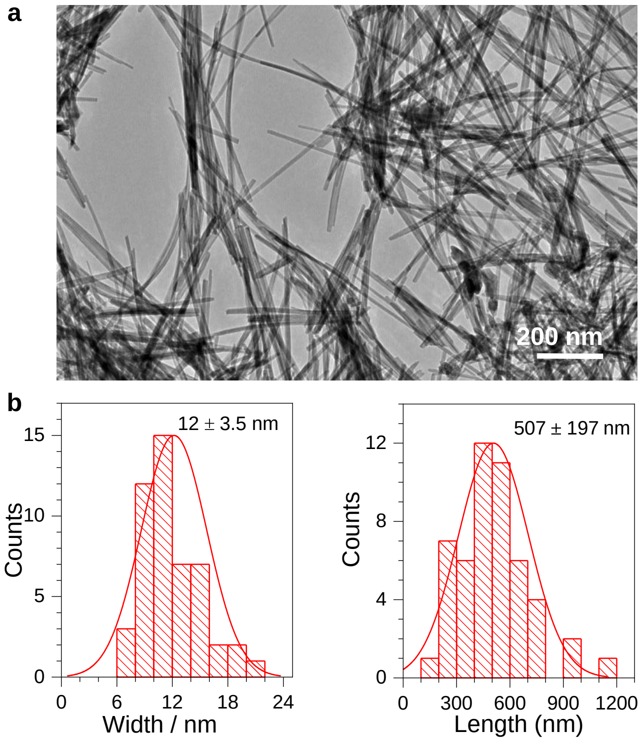

Figure S1. (a) TEM image of α-MnO_2 nanorods. (b) Width and length distributions of α-MnO_2 nanorods.

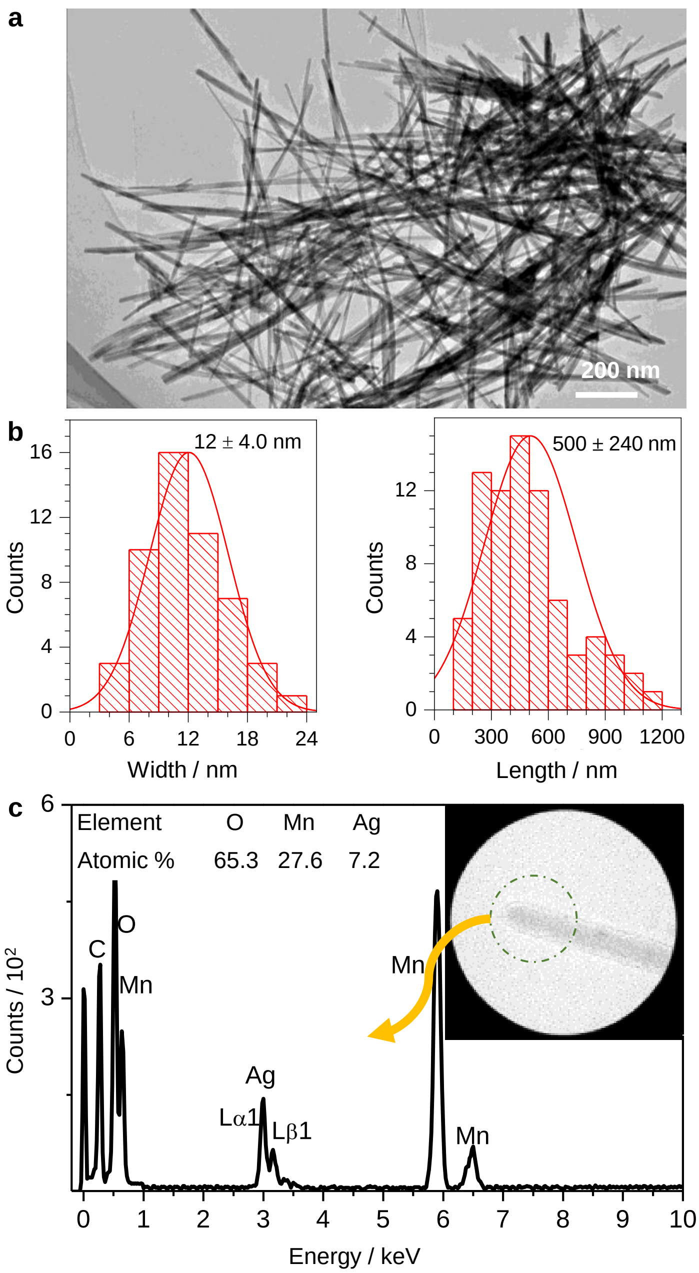

Figure S2. (a) TEM image of ASWs in α-MnO_2 tunnels. (b) Width and length distributions of ASWs. (c) Energy-dispersive X-ray spectroscopy and the corresponding elemental composition (inset table) of ASWs in α-MnO_2 tunnels (inset TEM image of an individual nanowire analyzed by EDX).

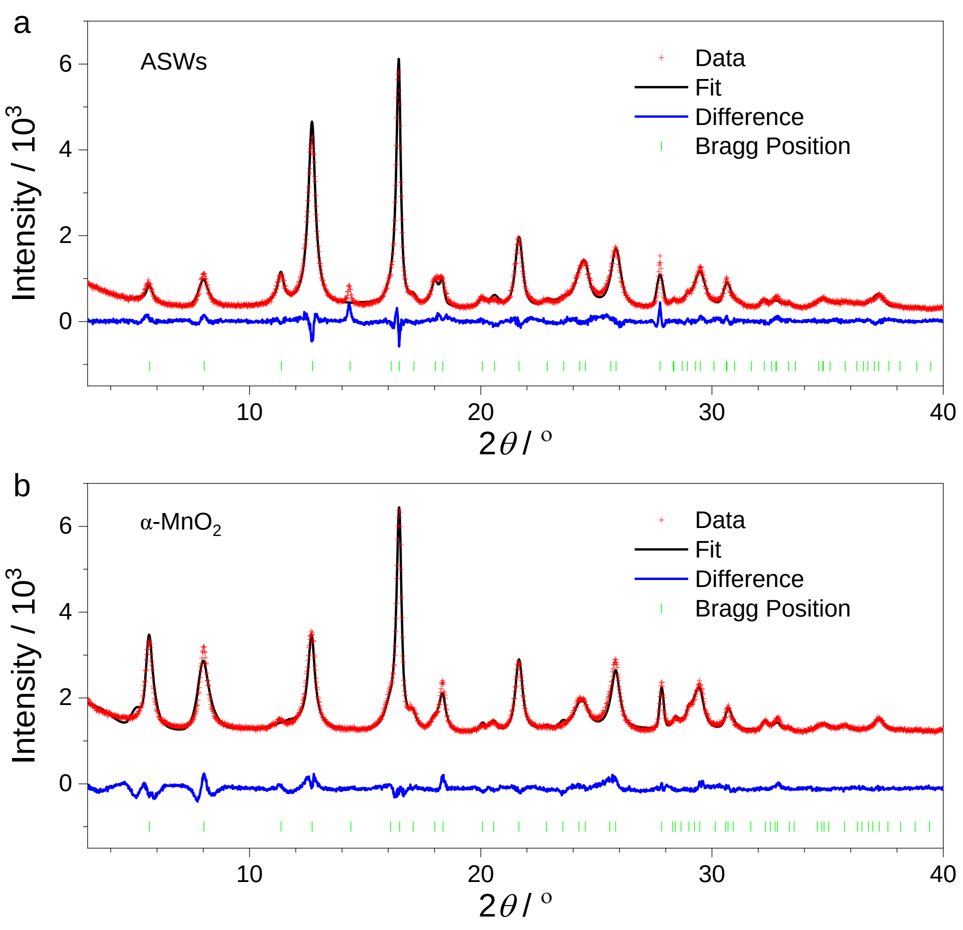

Figure S3. Room-temperature SXRD patterns of (a) the α-MnO_2 and (b) ASWs inside the α-MnO_2 tunnels. The short vertical lines below the SXRD mark the peak positions of all the Bragg reflections of the Hollandite manganese oxide with a tetragonal structure and an *I*4/*m* space group.

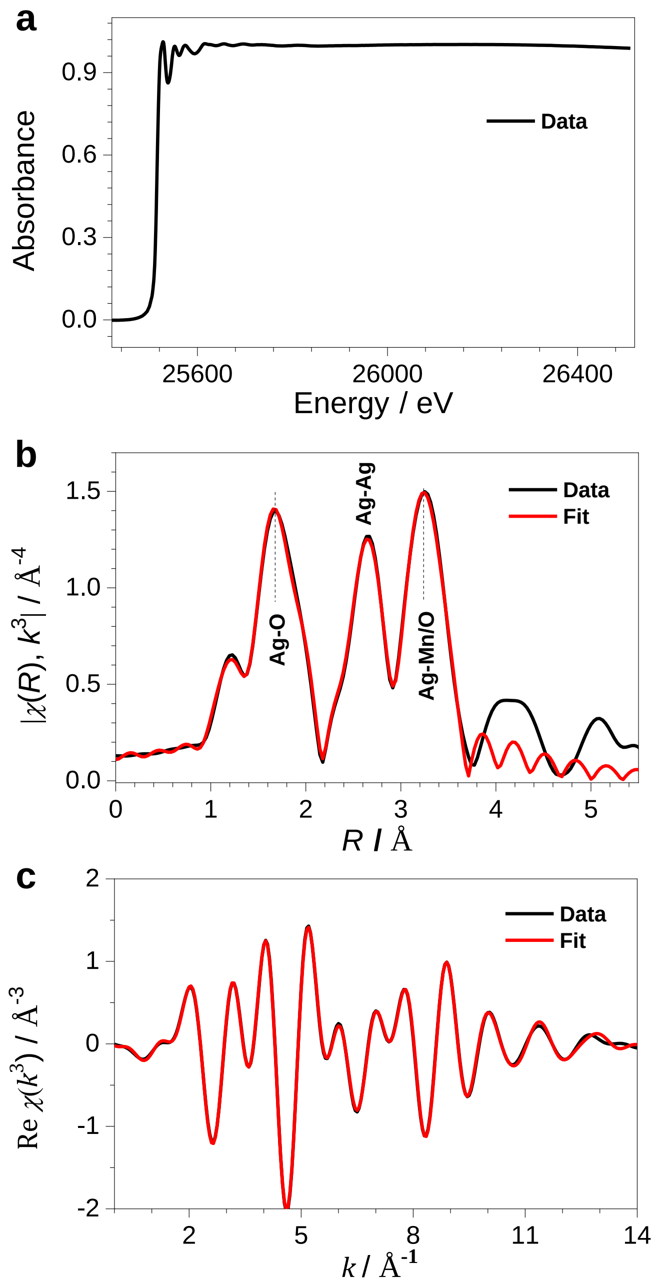

Figure S4. (a) Room-temperature Ag *K*-edge X-ray absorption spectrum, (b) the corresponding Ag *K*-edge χ(R) k^3-weighted FT EXAFS, (c) and the inverse FT EXAFS spectra of ASWs. Δk = 2.1-12.5 Å^{-1}, Δr = 1.3-3.8 Å.

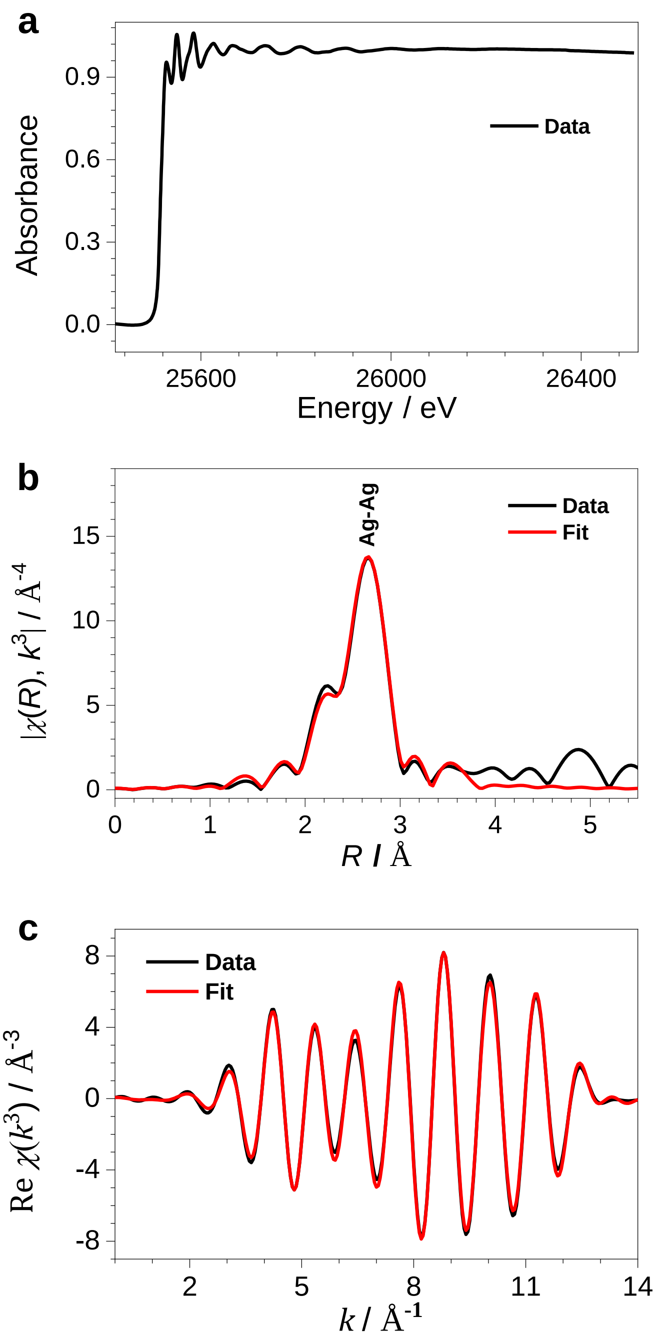

Figure S5. (a) Room-temperature Ag *K*-edge X-ray absorption spectrum, (b) the corresponding Ag *K*-edge χ(R) k^3-weighted FT EXAFS, (c) and the inverse FT EXAFS spectra of Ag foil. Δk = 2.1-12.5 $Å^{-1}$, Δr = 1.3-3.8 Å.

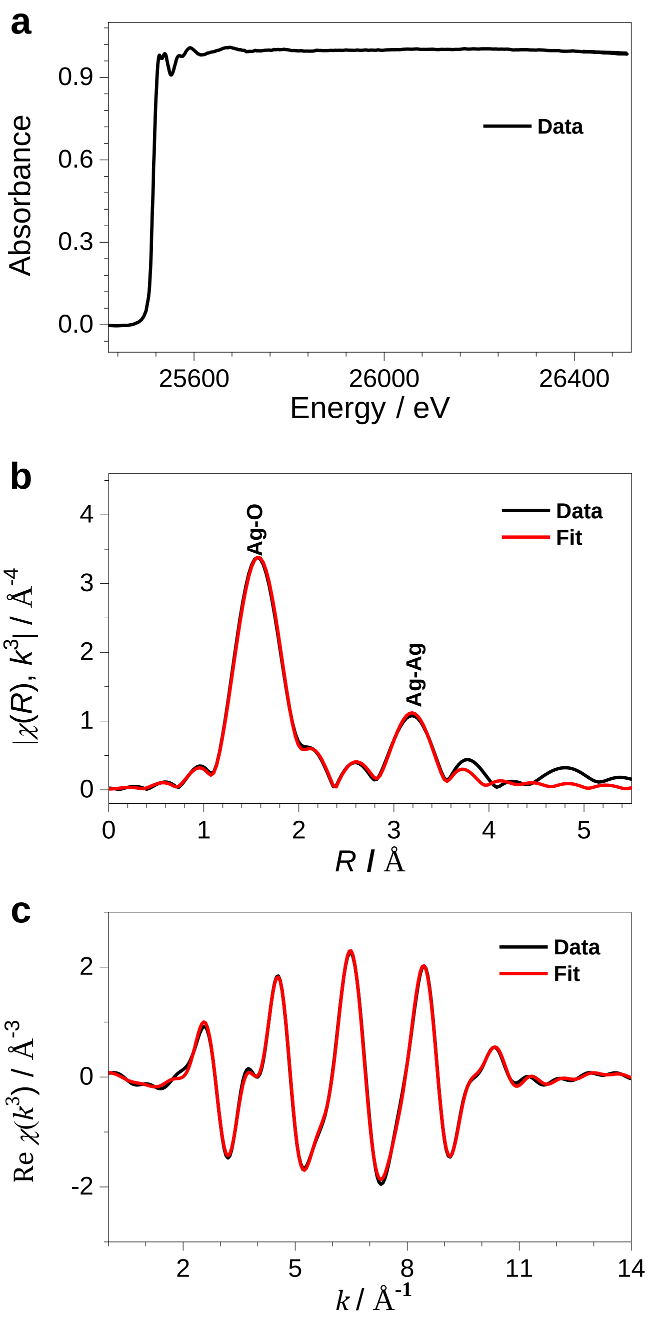

Figure S6. (a) Room-temperature Ag *K*-edge X-ray absorption spectrum, (b) the corresponding Ag *K*-edge $\chi(R)$ k^3-weighted FT EXAFS spectra, (c) and the inverse FT EXAFS spectra of Ag_2O. Δk = 2.1-10.5 Å^{-1}, Δr = 1.2-3.9 Å.

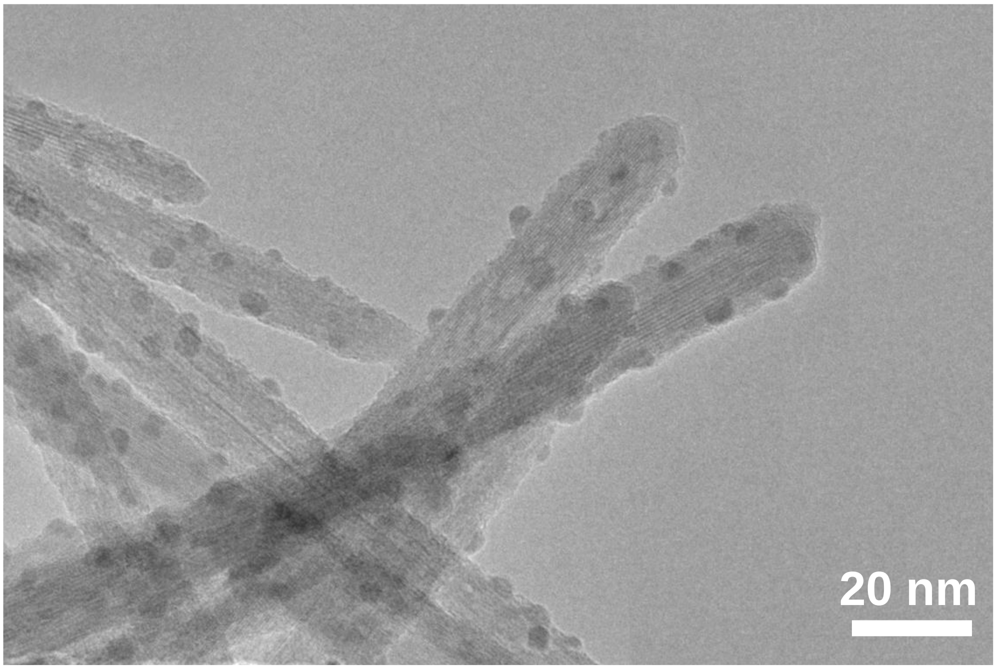

Figure S7. TEM image of Ag/MnO_2.

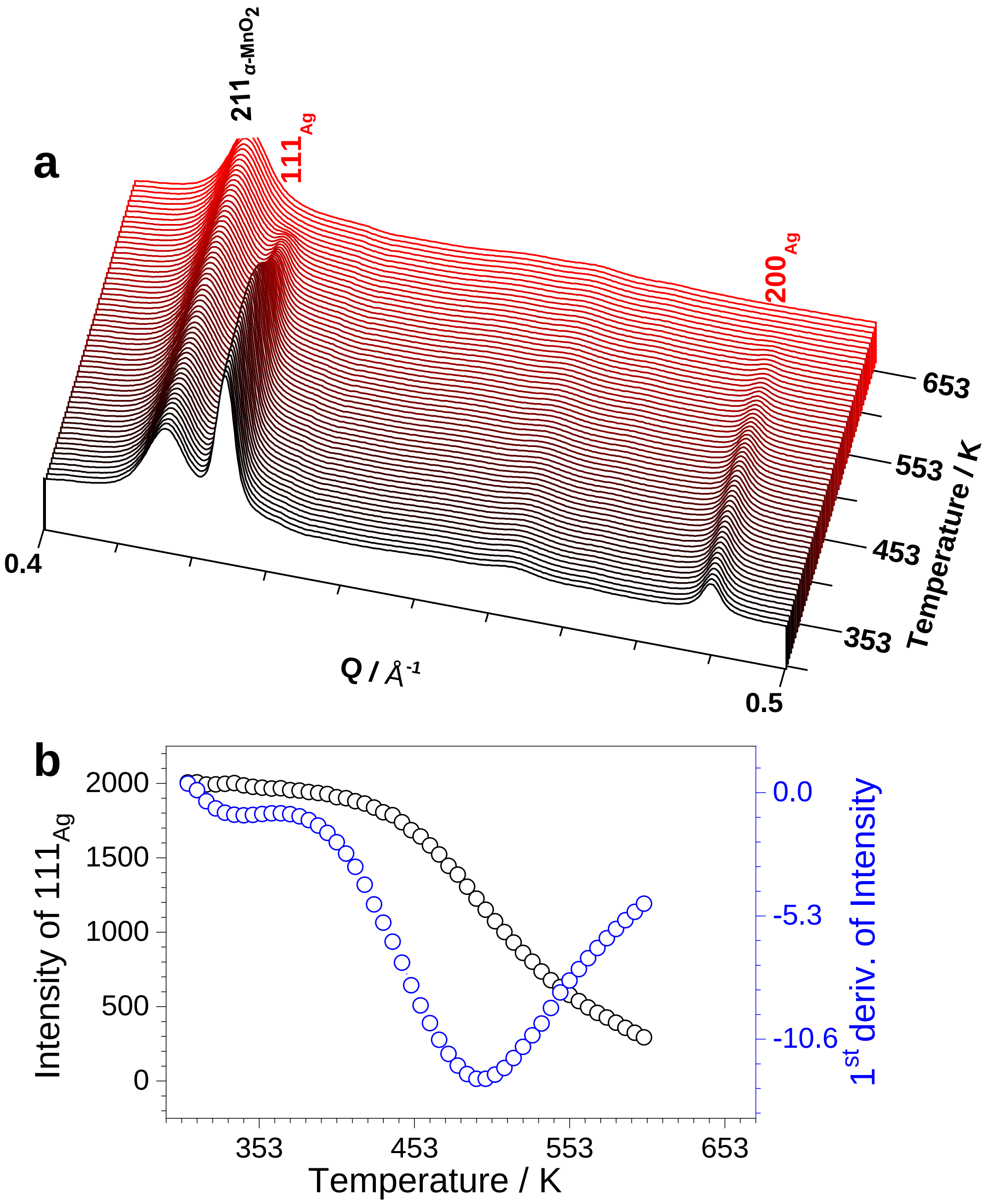

Figure S8. (a) Temperature-programmed XRD patterns of Ag/MnO_2 as a function of temperatures. The gradual change of the color from black to red indicates the gradual increase of temperature. (b) The Ag(111) intensity and the corresponding first derivative of the XRD patterns of Ag/MnO_2.

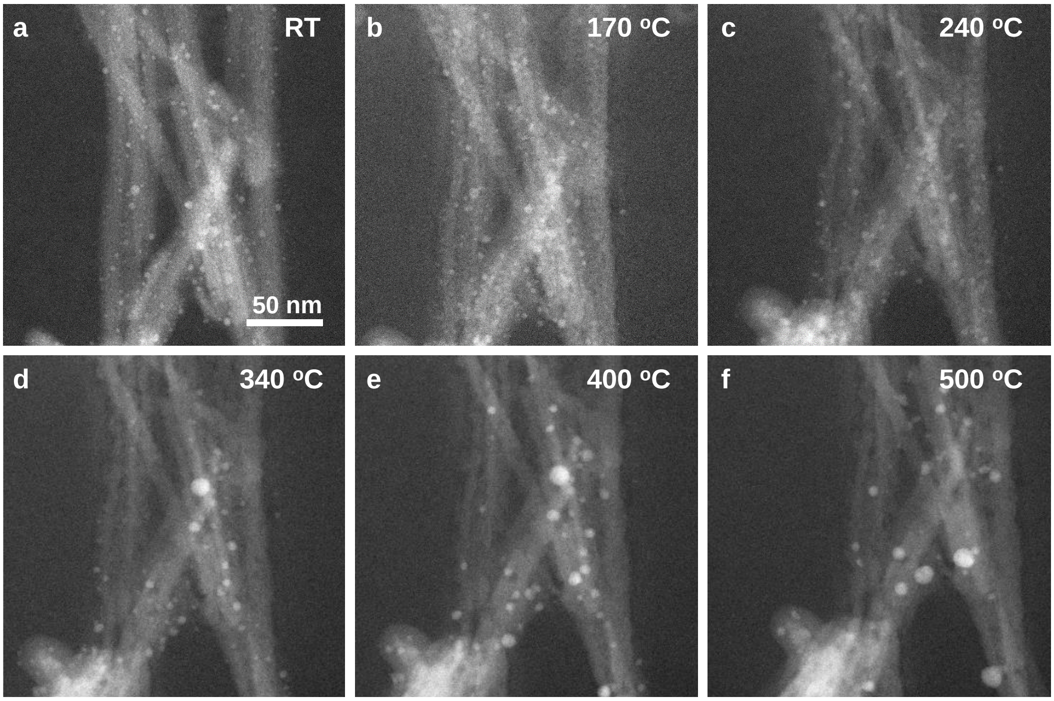

Figure S9. HAADF-STEM images of Ag/MnO_2 recorded in situ at different temperatures during the heating treatment under atmospheric N_2 environment. The in situ data show that bright silver nanoparticles aggregated into larger nanoparticles.

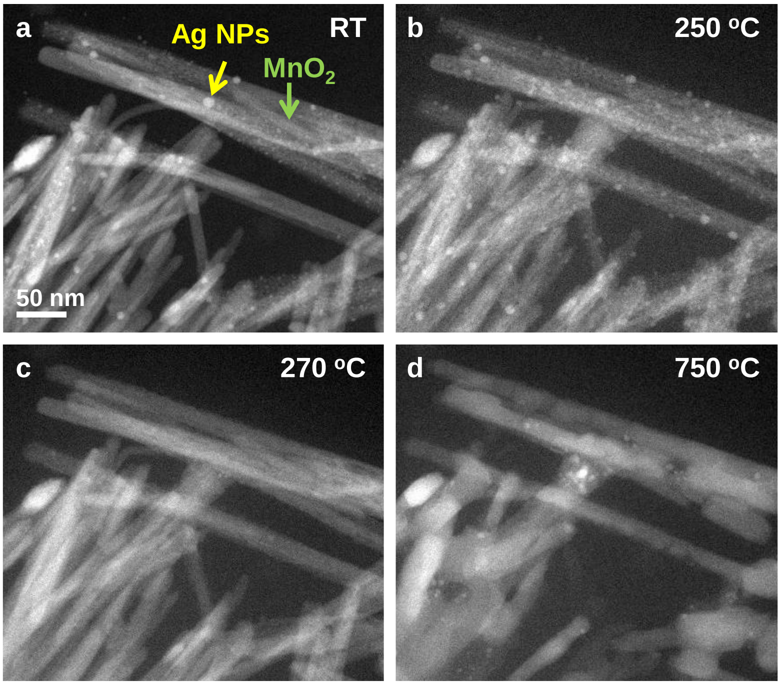

Figure S10. HAADF-STEM images of Ag/MnO_2 recorded in situ at different temperatures during the heating treatment under atmospheric O_2 environment. Clearly, all silver nanoparticles disappeared above 250 ^{o}C in the presence of O_2. In order to examine if silver atoms diffused into the matrix or immigrated to other places, the temperature was increased to 750 ˚C to destroy the structure of α-MnO_2. It can be seen that the morphologies of α-MnO_2 nanorods changed greatly and the hosted silver atoms were released and aggregated into larger nanoparticles again.

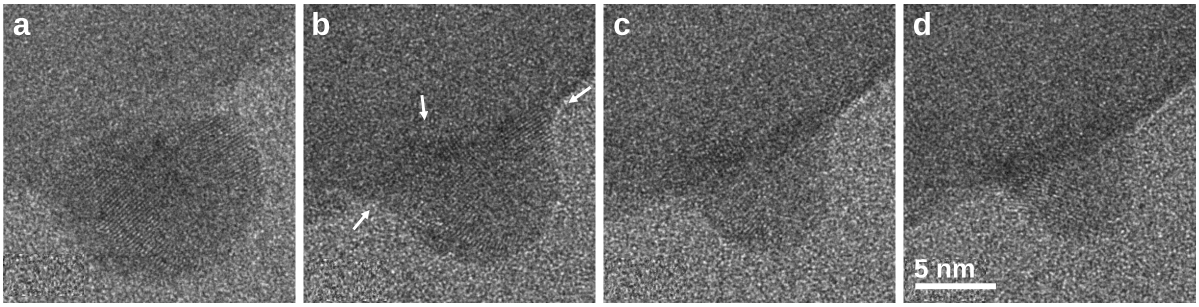

Figure S11. A serial of TEM images of Ag/MnO_2 recorded in situ at 270 ^oC in the presence of O_2 (see Supplementary Movie). Clearly, during the redispersion process, a stronger adherence of silver nanoparticle to α-MnO_2 was observed, leading to the collapse of the Ag nanoparticle. It looks like that the process is surface-mediated, in which atomic species after being emitted from a metal nanoparticle diffuse on the surface of the support until being trapped by a strong metal-support interaction.

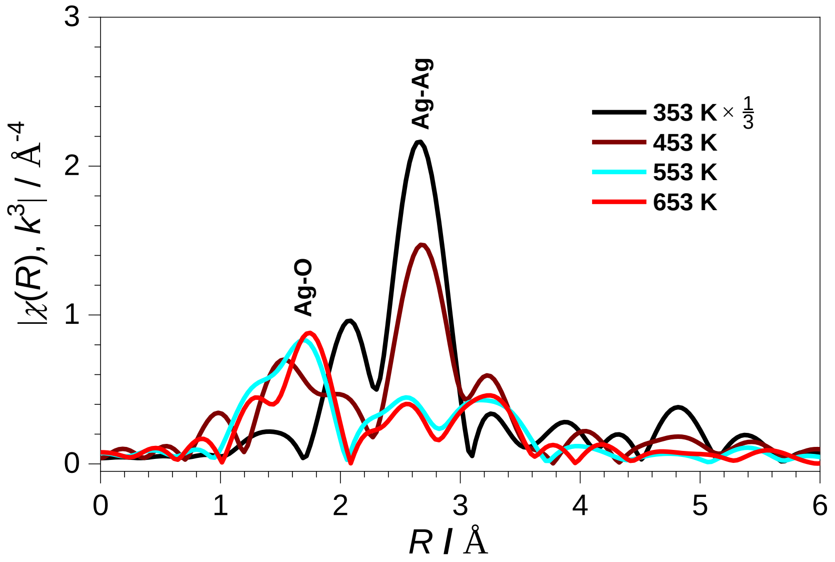

Figure S12. In situ Ag *K*-edge χ(R) k^3-weighted FT EXAFS spectra of Ag/MnO_2 measured at high temperatures.

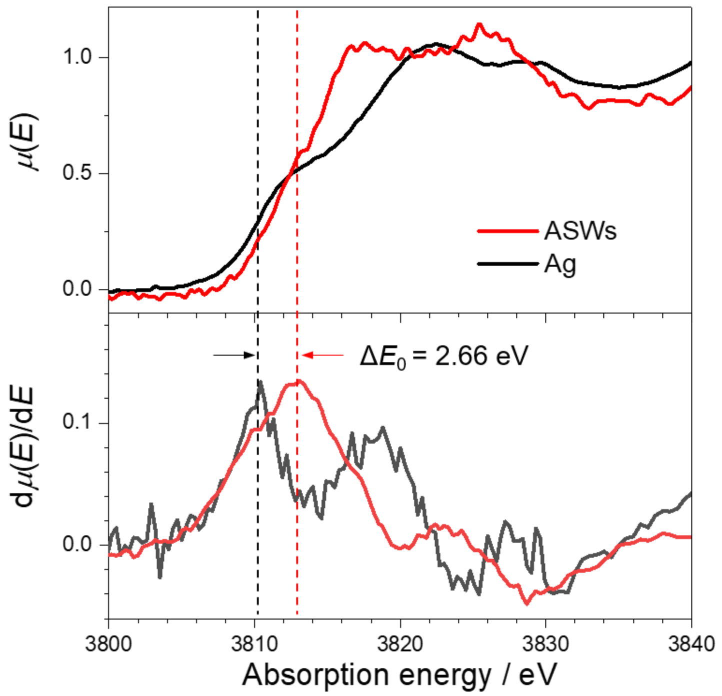

Figure S13. Ag L_1-edge XANES spectra and first derivative spectra of ASWs and the Ag reference sample.

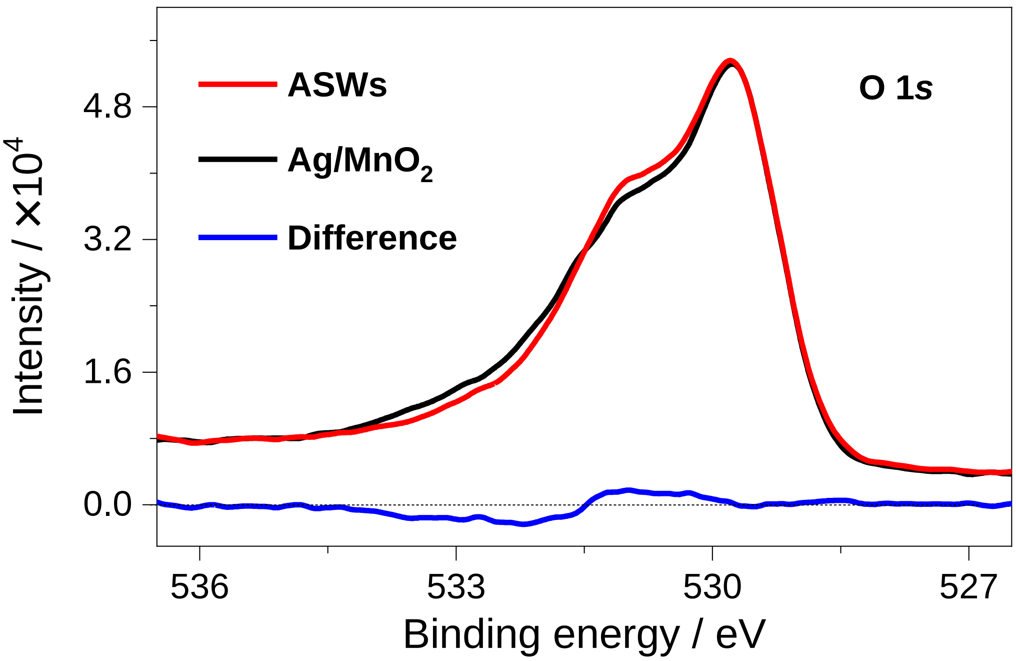

Figure S14. O 1*s* XPS of Ag/MnO_2 and ASWs in the α-MnO_2 tunnels together with their difference.

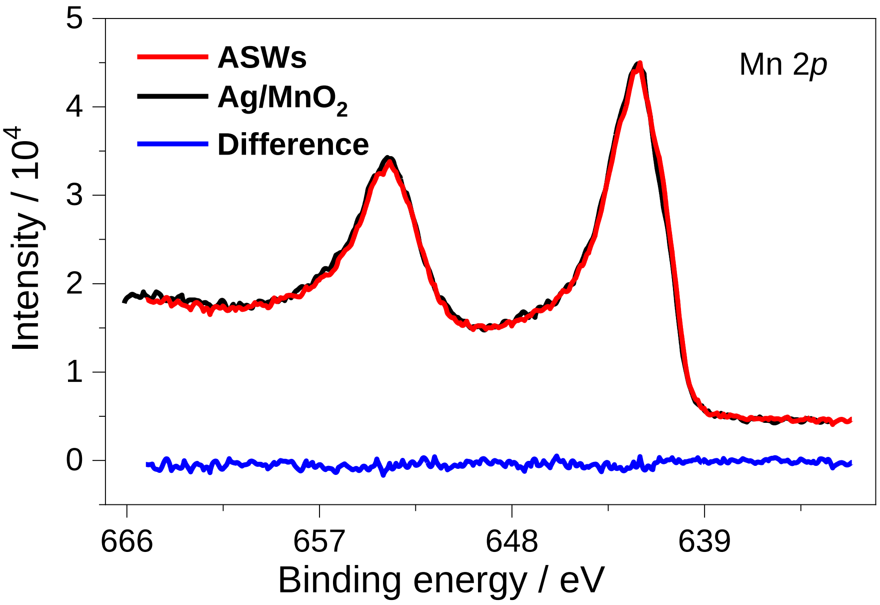

Figure S15. Mn 2*p* XPS of Ag/MnO_2 and ASWs in the α-MnO_2 tunnels together with their difference.

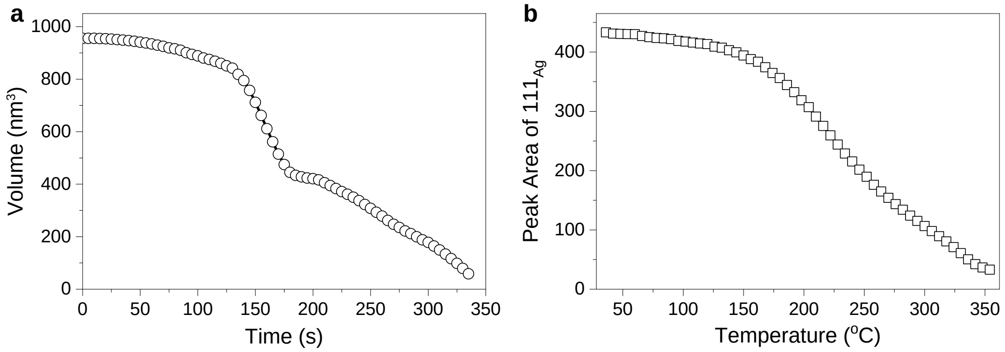

Figure S16. (a) The decrease in volume of the single Ag nanoparticle regarded as spherical as a function of calcination time (270 ^{o}C, atmospheric O_2 environment, also see Supplementary Figure 11 and Movie). (b) Integrated area of Ag_{111} peak as a function of calcination temperature (Air, 3 $^{o}C\ min^{-1}$).

3. Supplementary Tables

Table S1. Crystallographic data and details of the α-MnO_2 and (b) ASWs inside the α-MnO_2 tunnels in the data collections and the Rietveld refinements.

Samples	α-MnO_2	ASWs inside the α-MnO_2 tunnel
Chemical formula	MnO_2	$Ag_{0.13}MnO_2$
Crystal system	tetragonal	tetragonal
Space group	*I*4/*m*	*I*4/*m*
Z [a]	8	8
a / Å	9.8240(5)	9.8578(3)
c / Å	2.8617(2)	2.8758(8)
V / Å^3	276.2(3)	279.5(2)
R_p [b]/ %	5.254	5.138
R_{wp}[c]/ %	6.733	6.820
Wavelength / Å	0.826926	0.826926
2θ range / °	6-70	6-70
2θ step width / °	0.02	0.02

[a] *Z*, the number of MnO_2 and Ag_xMnO_2 formula units per unit cell;

[b] R_p, the unweighted profile factor;

[c] R_{wp}, the weighted profile factor.

Table S2. Structural parameters of the α-MnO_2 and (b) ASWs inside the α-MnO_2 tunnels.

Samples	Atom	*x*	*y*	*z*	occ[a]	beq[b]
α-MnO_2	Mn	0.3303(2)	0.1469(2)	0.5	1	1.5(2)
	Oi	0.2033(6)	0.1546(8)	0	1	0.8(1)
	Oii	0.1636(7)	0.5306(7)	0	1	0.4(1)
ASWs	Ag	0	0	0	1	3.0(3)
	Mn	0.1458(2)	0.3275(2)	0.5	1	0.8(2)
	Oi	0.1508(8)	0.2083(7)	0	1	1.3(2)
	Oii	0.1828(8)	0.4572(9)	0	1	0.5(2)

[a] occ, occupancy;

[b] beq, isotropic temperature factor (thermal parameter).

Table S3. EXAFS parameters of ASWs, Ag foil, and Ag_2O.

Sample	Shell	R (Å)	CN	σ^2 (Å^2)	ΔE_0 (eV)
ASWs	Ag-O	2.45	4.0	0.014	+6.4
	Ag-Ag	2.87	2.0	0.014	-2.6
Ag foil	Ag-Ag	2.86	12.0	0.011	+1.5
Ag_2O[a]	Ag-O	2.04	2.0	0.005	+4.3
	Ag-Ag	3.31	12.0	0.05	+5.4

R, distance between absorber and backscatter atoms;

CN, coordination number;

σ^2, Debye-Waller factor;

ΔE_0, energy shift;

R-space fit, Δk = 2.1-12.5 Å^{-1}, Δr = 1.3-3.8 Å.

[a] For Ag_2O, R-space fit, Δk = 2.1-10.5 Å^{-1}, Δr = 1.2-3.9 Å.
