# Supplementary material for: Stable single atomic silver wires assembling into a circuitry-connectable nanoarray
Source: Nat Commun. 2021 Feb 19;12:1191. doi: 10.1038/s41467-021-21462-3 (PMC7895918; doi:10.1038/s41467-021-21462-3)
Supplement: Supplementary file 4 — Description of Additional Supplementary Files [file 41467_2021_21462_MOESM4_ESM.pdf]

Title: Supplementary Movie:

Description: Dynamic structure of a Ag nanoparticle supported on α-MnO_2 at 270 ^{o}C in the presence of O_2.
